# Effect of 20(S)-Hydroxycholesterol on Multilineage Differentiation of Mesenchymal Stem Cells Isolated from Compact Bones in Chicken

**DOI:** 10.3390/genes11111360

**Published:** 2020-11-17

**Authors:** Roshan Adhikari, Chongxiao Chen, Woo Kyun Kim

**Affiliations:** 1 Department of Poultry Science, University of Georgia, 303 Poultry Science Building, Athens, GA 30602-2772, USA; roshan.adhikari@cj.net; 2 Prestage Department of Poultry Science, North Carolina State University, 2711 Founders Drive, Raleigh, NC 27695-7608, USA; sean_chen@ncsu.edu

**Keywords:** adipogenesis, chicken, mesenchymal stem cells, myogenesis, osteogenesis, 20(s)-hydroxycholesterol, hedgehog signaling

## Abstract

Bone health and body weight gain have significant economic and welfare importance in the poultry industry. Mesenchymal stem cells (MSCs) are common progenitors of different cell lineages such as osteoblasts, adipocytes, and myocytes. Specific oxysterols have shown to be pro-osteogenic and anti-adipogenic in mouse and human MSCs. To determine the effect of 20(S)-hydroxycholesterol (20S) on osteogenic, adipogenic, and myogenic differentiation in chicken, mesenchymal stem cells isolated from compact bones of broiler chickens (cBMSCs) were subjected to various doses of 20S, and markers of lineage-specific mRNA were analyzed using real-time PCR and cell cytochemistry. Further studies were conducted to evaluate the molecular mechanisms involved in lineage-specific differentiation pathways. Like human and mouse MSCs, 20S oxysterol expressed pro-osteogenic, pro-myogenic, and anti-adipogenic differentiation potential in cBMSCs. Moreover, 20(S)-Hydroxycholesterol induced markers of osteogenic genes and myogenic regulatory factors when exposed to cBMSCs treated with their specific medium. In contrast, 20S oxysterol suppressed expression of adipogenic marker genes when exposed to cBMSCs treated with OA, an adipogenic precursor of cBMSCs. To elucidate the molecular mechanism by which 20S exerts its differentiation potential in all three lineages, we focused on the hedgehog signaling pathway. The hedgehog inhibitor, cyclopamine, completely reversed the effect of 20S induced expression of osteogenic and anti-adipogenic mRNA. However, there was no change in the mRNA expression of myogenic genes. The results showed that 20S oxysterol promotes osteogenic and myogenic differentiation and decreases adipocyte differentiation of cBMSCs. This study also showed that the induction of osteogenesis and adipogenesis inhibition in cBMSCs by 20S is mediated through the hedgehog signaling mechanism. The results indicated that 20(S) could play an important role in the differentiation of chicken-derived MSCs and provided the theory basis on developing an intervention strategy to regulate skeletal, myogenic, and adipogenic differentiation in chicken, which will contribute to improving chicken bone health and meat quality. The current results provide the rationale for the further study of regulatory mechanisms of bioactive molecules on the differentiation of MSCs in chicken, which can help to address skeletal health problems in poultry.

## 1. Introduction

The popularity of modern broiler meat-type chicken has grown tremendously in size in the last 60 years compared to its ancestors. However, with advanced genetic selection and increased production efficiency, several welfare problems such as increased leg and skeletal disorders, woody breast, and high-fat mass are challenges for the broiler industry [1,2,3]. Genetic selection has provided an efficient increase in growth rate and meat yield every year. However, support systems such as skeletal and cardiovascular system have not kept up with the increasing body mass, making modern broiler chicken susceptible to several welfare problems that create economic problems for the poultry industry [4]. Similarly, laying hens and breeders suffer from osteoporosis, cage layer fatigue, keel bone damage, resorption of cortical bone, increased medullary bone mass, and high abdominal fat deposition during egg production [5,6]. Modern laying hen strains are under negative calcium balance as soon as they enter the laying phase [7], when there is a massive demand for calcium during egg formation and a rapid turnover of medullary bones during egg production [8]. Such a process increases osteoporosis, brittle bone, breakage of the keel bone, tibia dyschondroplasia, and other skeletal problems in laying hens and breeders [9,10]. Changes in the housing system and the introduction of aviary and enriched cage systems have increased skeletal issues such as keel bone fracture in modern laying hens [11,12,13]. Bone problems are still among the costliest health disorders for the poultry industry, both in meat and egg type birds. They result in reduced feed intake, decreased weight gain, lower egg production, decreased production efficiency, increased lameness and reduced feet quality, and increased frequency of downgrades and condemnations when affected birds are processed. Bone abnormality is reported to be initiated by developmental, degenerative, nutritional, environmental, and infectious problems and this could be due to a combination of one or more of these causes [4].

Mesenchymal Stem cells derived from compact chicken bones (cBMSCs) are multipotent cells which can be a potential MSC-model to understand and address the skeletal and adipogenic health issues associated with broilers, laying hens, and breeders. Osteogenic and adipogenic cells are reported to have opposite regulations. Reduction in the number of osteoblastic cells increased bone resorption by osteoclasts, and osteoblast shift towards adipogenic cells in aging and osteoporotic cells could have caused the change in skeletal problems [14]. In various cases of osteoporosis, it has been reported that the number of adipocyte cells increases with a decrease in both the number of osteoblasts and the proliferation capacity of osteoprogenitor cells [15,16]. Similarly, bone loss in age-related osteoporosis could be due to the shifting of mesenchymal stem cell (MSCs) differentiation into the adipogenic pathway rather than the osteogenic pathway [17,18].

MSCs are multipotent cells with high proliferation and differentiation capabilities that can differentiate into several tissue lineages such as adipocytes, osteoblasts, chondrocytes, myocytes, and fibroblasts [19,20,21,22]. MSCs are isolated from various tissue origins such as bone, adipose tissue, cord blood, placenta, as well as many other tissues [19,22,23,24,25,26]. MSCs isolated from bone have been reported to differentiate into several different progenitor cells, of which osteoblasts and adipocytes share a potential, reciprocal relationship in differentiation [27,28]. A decrease in bone formation and an increase in adipogenesis in the bone marrow could increase osteoporosis and bone fracture [29]. The decline in bone synthesis due to reduced osteoblast formation from progenitor cells, and increased adipocyte formation at the expense of those osteoblasts due to age or disease could lead to fragile bone and fracture [30,31]. The differentiation fate of a MSCs population is determined by the interplay of complexed extracellular mediators such as hormones, growth factors, nutrients, and pharmacological agents that affect the molecular mechanisms and signaling pathways of lineage-specific transcription factors. Regulation of stem cell fate down these various lineages is essential for tissue development, homeostasis, and repair [32]. MSCs derived from humans and mice have been used as a useful tool for studying the mineralization and differentiation potential of MSCs towards osteogenic lineage. MSCs have also been isolated from chicken bone marrow, lungs, cartilage, adipose tissue in previous studies [33,34,35,36,37]. However, there is limited information available to understand the multilineage differentiation potential and pathways of cBMSCs when subjected to possible osteogenic/adipogenic bioactive compounds.

Oxysterols are oxygenated derivatives of cholesterol oxidation, a 27-carbon molecule present in the circulation in human and animal tissue [38]. Oxysterol is involved in many different physiological, biological, and pathological roles in animals such as cholesterol efflux, lipoprotein metabolism, calcium uptake, atherosclerosis, and apoptosis [39]. It has been reported that oxysterol plays a possible role in regulating cellular differentiation of MSCs. Different oxysterol, such as 20 (S)-hydroxycholesterol (20S), alone or in combination with 22 (S) or 22 (R)-hydroxycholesterol exert pro-bone and anti-fat effects in M2-10B4 mouse bone marrow stromal cells [40]. Further studies have described that 20S oxysterol stimulated differentiation of pluripotent bone marrow stem cells derived from mouse and humans by activating hedgehog signaling in vitro [41]. Some oxysterols are potential bioactive molecules capable of suppressing adipogenic differentiation in mouse and human MSCs [42]. Oxysterols are derivatives of the endogenous cellular cholesterol biosynthesis pathway, and this pathway is essential in the osteogenic differentiation of progenitor cells. Thus, oxysterol could act as an osteoinductive compound [43,44]. Studies on mouse and human MSCs can be conducted to understand the effect of oxysterol, a potential bioactive compound on multilineage differentiation of cBMSCs.

There is a lack of research on understanding the combination of developmental, regenerative, and nutritional aspects to address poultry’s skeletal health. Research on the skeletal health of poultry has only focused on Ca, P, and vitamin D metabolism. Studies on the effect of potentially bioactive compounds such as 20S in differentiation and the pathways involved in the differentiation of cBMSCs can help address the skeletal and adipogenic poultry issues. This study aimed to understand the effect of 20S on osteogenic, adipogenic, and myogenic differentiation potential of cBMSCs and examine the possible role of hedgehog signaling pathway in multilineage differentiation in cBMSCs.

## 2. Materials and Methods

### 2.1. Isolation of Compact Bone-Derived Mesenchymal Stem Cells

All experiments and procedures involving birds were performed following the guidelines for the use of animals in research stated by the Animal Care and Use Committee at the University of Georgia (A2018 09-013). MSCs were obtained from compact bones of tibia and femur of day-old broiler chicken following a modified protocol described earlier for MSCs derived from mouse compact bone [45]. Two broiler chicks were aseptically dissected, and the legs were collected. Bone marrow was flushed with phosphate-buffered saline (PBS) containing 2% FBS (Fisher scientific, Hampton, NH, USA), and compact bone was chopped into small pieces. Bones were digested with 10 mL Dulbecco’s Modified Eagle’s Medium (DMEM) (Mediatech Inc, Manassas, VA, USA) digestion buffer containing 0.25% collagenase (Sigma-Aldrich, St. Louis, MO, USA) and 20% FBS for 55 min in a shaker at 180 rpm and 37 °C. Contents were filtered in 100 mm sterile nylon meshes (Fisher scientific, USA) and centrifuged at 1200× *g* rpm for eight min. The supernatant was discarded, and cell pellets were suspended in 10 mL DMEM containing 10% fetal bovine serum (FBS) (GE Healthcare, Chicago, IL, USA), 100 U/mL penicillin, 100 µg/mL streptomycin, and L-glutamate (Mediatech, Manassas, VA, USA). Cells were then seeded into 100 mm Petri plates (BD Bioscience, San Jose, CA, USA) and cultured at 37 °C in a 5% CO_2_ incubator. Half of the media was replaced by fresh media at 12 h, and complete media was changed at 24 h to remove the non-adherent cells. After that, the media was replaced every three days until confluent.

### 2.2. Cell Culture

Cells were expanded in 10 mL DMEM containing 10% FBS, 100 U/mL penicillin, 100 µg/mL streptomycin, and L-glutamate. Once cells were confluent, cells were washed twice with 5 mL PBS, trypsinized with 1X Trypsin Ethylenediaminetetraacetic acid (TE) (Mediatech, Manassas, VA, USA) at 37 °C for two min. Eight ml of 10% DMEM media was added to the petri plate. Cells were washed to remove any adherent cells and transferred to a centrifuge tube. The contents were centrifuged at 1200× *g* rpm for eight min; the supernatant was discarded, and cell pellets were plated at a rate of 20,000 cells/cm^2^. All experiments were conducted between passages 4 and 5. A series of experiments were conducted to understand the effect of 20S in osteogenic, myogenic, and adipogenic differentiation of cBMSCs isolated from day-old broiler compact bones. Confluent cBMSCs were plated in 6-well plates and treated with different levels of assigned treatments. Confluent cells were treated with varying levels of 20S along with respective differentiation media. All experiments were repeated twice to validate the results.

### 2.3. Osteogenic Differentiation of cBMSCs

Two experiments were conducted to understand the effect of 20S on osteogenic differentiation of cBMSCs. In the first experiment, at passage four, confluent cBMSCs were treated with one of five treatments; (1) Control (C), (2) osteogenic media (OM) containing DMEM with 10% FBS, 50 ug/mL ascorbate, 0.5 µM dexamethasone (DXA), and 10 mM β-glycerophosphate, (3) OM + 2.5 µM 20S, (4) OM + 5 µM 20S, and (5) OM + 10 µM 20S. Cells treated in 6-well plates were harvested at 24 h, 72 h, and 7 d to examine osteogenic mRNA transcripts using quantitative reverse transcription polymerase chain reaction (qRT-PCR). Cell cytochemistry (Von Kossa and Alizarin Red) was conducted on cells cultured in 24-well plates (Greiner bio-one, Kremsmünster, Austria) at 7 d and 14 d to observe osteogenic differentiation of cells.

The second experiment was conducted to determine whether Hedgehog (Hh) signaling pathway was involved in the molecular mechanism of osteogenic differentiation of cBMSc when treated with 20S. Confluent cells were pretreated with either control vehicle (Veh) or with 4 µM of Hh signaling pathway inhibitor cyclopamine (Cyc) for 2 h followed by one of four treatments: (1) C, (2) osteogenic media (OM) containing DMEM with 5% FBS, 50 ug/mL ascorbate, 0.1 µM DXA, and 10 mM β-glycerophosphate, (3) OM + 10 µM 20S, or (4) 10 µM 20S alone, for a total of eight treatment combinations. Cells treated with or without Cyc in 6-well plates were harvested at 24 h and 72 h to understand osteogenic mRNA through qRT-PCR.

### 2.4. Adipogenic Differentiation of cBMSCs

The third experiment was conducted to understand the dose-effect of 20S in the adipogenic differentiation of cBMSCs. Confluent cells at passage 4 were treated with one of five treatments: (1) control (C), (2) 300 µM Oleic Acid (OA), (3) OA + 2.5 µM 20S, (4) OA + 5 µM 20S, or (5) OA + 10 µM 20S for 12, 24, 48, and 96 h. Cells cultured in 6-well plates were harvested for mRNA expression of adipogenic transcripts using qRT-PCR at 12 h, 24 h, 48 h, and 96 h. Cells were stained with Oil Red O stain to visualize lipid deposition at 48 h.

The fourth set of experiments was conducted to evaluate whether the anti-adipogenic effects of the 20S are mediated through the hedgehog signaling pathway. Confluent cells were pretreated with Veh or Cyc for 2 h. Each pretreated group of cells was treated with C, 300 µM OA, 10 µM 20S, and 300 µM OA + 10 µM 20S for 24 h and 48 h. Samples in 6-well plates were harvested at 24 h and 48 h to understand osteogenic mRNA transcripts using qRT-PCR.

### 2.5. Myogenic Differentiation of cBMSCS

The fifth set of experiments was conducted to understand the effect of 20S in the myogenic differentiation of cBMSCs. cBMSCs at passage four were treated with one of five treatments: (1) C, (2) myogenic media (MM) containing DMEM with 5% Horse Serum, 50 µM Hydrocortisone, and 0.1 µM DXA, (3) MM + 2.5 µM 20S, (4) MM+ 5 µM 20S, or (5) MM + 10 µM 20S. Cells in 6-well plates were harvested for evaluating mRNA expression of adipogenic transcripts using quantitative reverse transcription polymerase chain reaction (qRT-PCR) at 24 h, 72 h, 7 d, and 14 d.

The sixth set of experiments was conducted to evaluate Hh signaling pathway’s involvement in myogenic differentiation of cBMSCs when treated with 20S. Cells were pretreated with Veh or with Cyc for 2 h. Each pre-treated group of cells was then treated with one of four treatments: C; MM; 10 µM 20S, and MM + 10 µM 20S. Samples in 6-well plates were harvested at 24 h and 72 h to analyze myogenic mRNA transcripts using quantitative reverse transcription polymerase chain reaction (qRT-PCR).

### 2.6. Adipocyte Differentiation—Oil Red O Stain

Oil Red O stain was performed on harvested cells at 48 h to detect adipocyte differentiation following the previously described protocol [39,46]. In brief, cells were washed and rinsed with PBS and fixed in 60% isopropanol for two min. Cells were stained with 0.4% Oil Red O stain in 100% isopropanol for 30 min and rinsed with double-distilled H2O. After staining, cells were visualized and photographed with a microscope for red lipid droplets.

### 2.7. Mineralization of Osteoblasts—Alizarin Red

Alizarin Red test was used to detect the mineralization of osteoblasts which produces a bright orange-red color when exposed to the Alizarin red solution (Sigma Aldrich, St. Louis, MO, USA). Alizarin red test was conducted following a modified protocol, as previously described [47]. In brief, Alizarin Red Stain solution was prepared to dissolve 1 mg of Alizarin Red S in 50 mL distilled water, and pH was adjusted to 4.1–4.3 with 0.1% NH_4_OH solution. Cells were fixed using 10% buffered formalin for 30 min and washed 4 times with distilled water. Cells were stained with Alizarin Red solution for 45 min in the dark. Cells were washed to remove the excess dye. PBS was added after washing and observed in a microscope for calcified orange/red staining.

### 2.8. Osteogenic Differentiation and Calcium Deposition—Von Kossa Stain

Von Kossa stain was used to detect the osteogenic differentiation and calcium deposition in differentiated cells. Matrix mineralization in cell monolayers was detected by silver nitrate staining, as previously described [48]. In brief, cells were washed twice with PBS, treated with 1% glutaraldehyde, and incubated for 15 min. Cells were washed with deionized water, and 5% silver nitrate was added to the cells. The cells were incubated in the dark for 30 min, washed twice with double distilled water, air dried, and exposed to bright light until black spots were developed in calcification areas.

### 2.9. Quantification of mRNA Expression Using qRT-PCR

At the specific time point of each experiment, cells were harvested, and total RNA was isolated using Qiazol reagent (Qiagen, Hilden, Germany) following the manufacturer’s protocol. Two µg of RNA was reverse transcribed using high capacity cDNA reverse transcription synthesis kits (Applied Biosystem, Atlanta, GA, USA) following the manufacturer’s protocol. Primers for each gene were designed and checked for target identity using the National Centre for Biotechnology Information (NCBI). To understand the possible alteration in expression of specific transcripts in the harvested cells, qRT-PCR analysis was performed using nuclease-free water, the forward and reverse primers of each particular target gene, template cDNA, and SYBR Green (Bio-Rad, Hercules, CA, USA) using StepOne^TM^ Real-Time PCR systems (Applied Biosystems, Foster City, CA, USA). Temperature cycles were as follows: 95 °C for 10 min followed by 40 cycles at 95 °C for 15 s, annealing temp for 20 s, and extension 72 °C for one minute. All analyses were done in duplicate reaction, and Glyceraldehyde 3-phosphate dehydrogenase (GAPDH) was used as a housekeeping gene for cu. After 40 cycles of qRT-PCR, melt curves were examined to ensure primer specificity. Fold changes in gene expression were calculated using the −ΔΔCt method and reported as fold changes of the target genes’ expression in experimental groups compared to the control group. Details of primers sequences used for the experiment are presented in Table 1.

### 2.10. Statistical Analysis

Data obtained were analyzed using the general linear model procedure of the Statistics Analysis System (SAS) Institute. Tukey test was used to measure the mean separation when statistically significant. Results were analyzed and presented as means ± SEM. The level of significance used in all three studies was a probability value of *p* < 0.05.

## 3. Results

### 3.1. Oxysterol Induced Osteogenic Differentiation of cBMSCs

**Figure 1:**
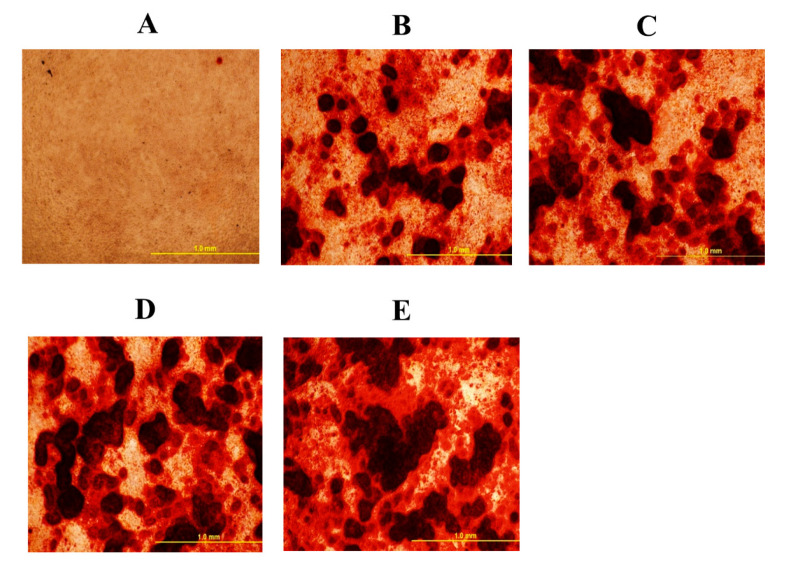
Mineral deposition in mesenchymal stem cells isolated from chicken compact bone stem cells treated with different levels of 20S oxysterol for 14 days (detected by alizarin red test). Calcium deposition was increased in cells treated with 20S oxysterol compared to control cells. Cells were treated with (**A**) Control (**B**) osteogenic media (OM) containing DMEM with 10% FBS, 50 µg/mL ascorbate, 0.5 µM DXA, and 10 mM β-glycerophosphate, (**C**) OM + 2.5 µM 20S, (**D**) OM + 5 µM 20S, or (**E**) OM + 10 µM 20S. Pictures were taken by Olympus DP70 at 4X.

**Figure 2:**
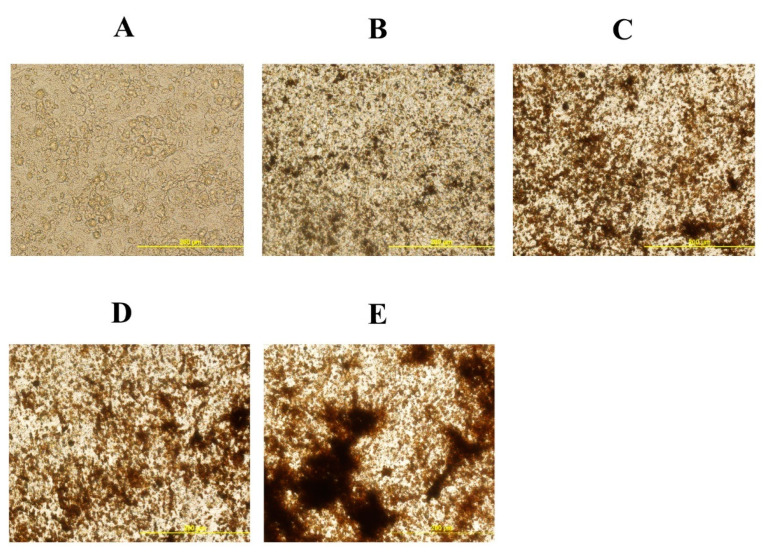
Mineral deposition in mesenchymal stem cells isolated from chicken compact bone stem cells treated with different levels of 20S oxysterol for 14 days (detected by Von Kossa stain). Calcium deposition was increased in cells treated with 20S oxysterol compared to control cells. Cells were treated with (**A**) Control, (**B**) osteogenic media (OM) containing DMEM with 10% FBS, 50 µg/mL ascorbate, 0.5 µM DXA, and 10 mM β-glycerophosphate, (**C**) OM + 2.5 µM 20S, (**D**) OM + 5 µM 20S, or (**E**) OM + 10 µM 20S. Pictures were taken by Olympus DP70 at 20X.

**Figure 3:**
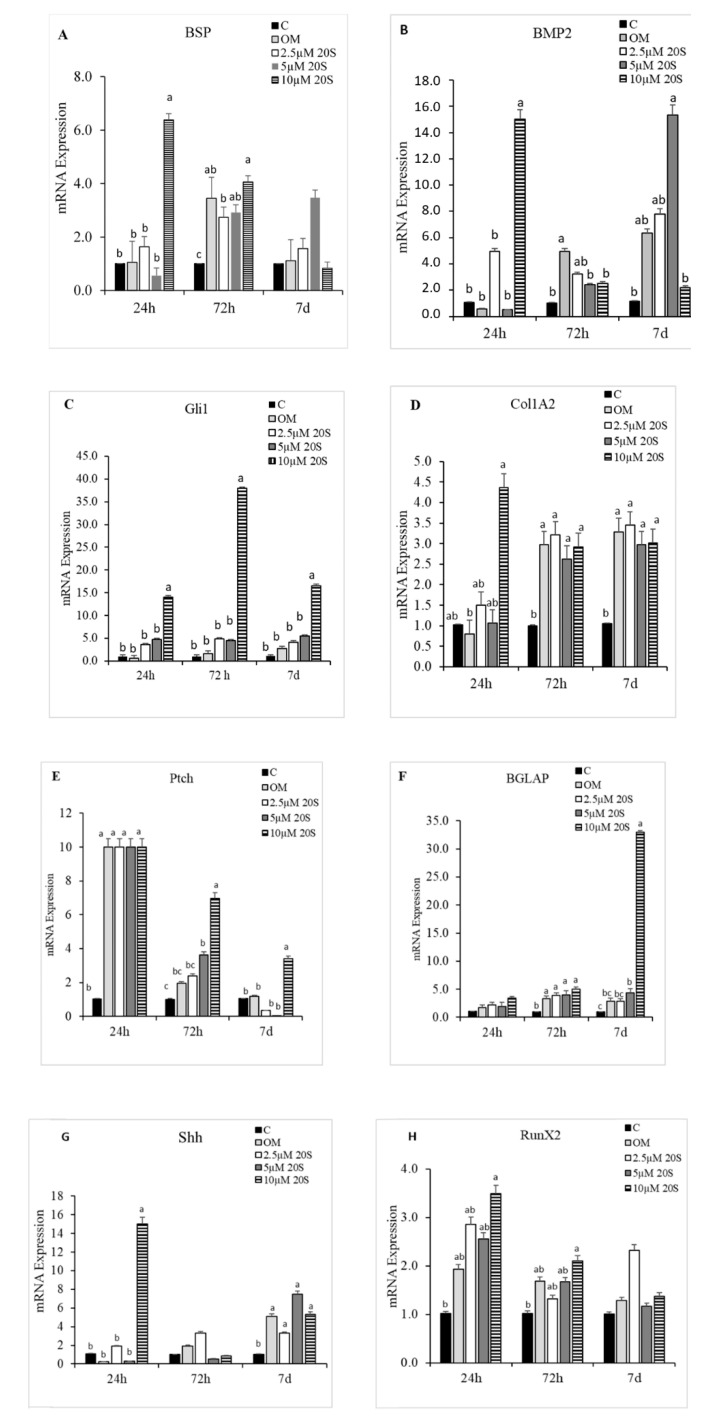
Osteogenic effects of 20S oxysterol in mesenchymal stem cells. Relative levels of mRNA expression of osteogenic genes (**A**) BSP, (**B**) BMP2, (**C**) Gli-1, (**D**) Col1A2, (**E**) Ptch, (**F**) BGLAP, (**G**) Shh, and (**H**) RunX2 in chicken compact bone-derived mesenchymal stem cells (cBMSCs) treated with different levels of oxysterol. cBMSCs were treated with (1) Control (C), (2) osteogenic media (OM) containing DMEM with 10% FBS, 50 µg/mL ascorbate, 0.5 µM DXA, and 10 mM β-glycerophosphate, (3) OM + 2.5 µM 20S, (4) OM + 5 µM 20S, or (5) OM + 10 µM 20S. Cells were harvested at 24 h, 72 h, and 7 d after treatment. RNA was isolated, reverse transcribed to cDNA, and qRT-PCR was conducted to analyze osteogenic gene expression. GAPDH was used as a housekeeping gene. Fold changes in gene expression relative to the control were calculated using the −ΔΔCt method. Bars represent mean ± SEM for triplicate determinations. Within each harvested period, bars without a letter (a–b) in common are significantly different (*p* < 0.05) when analyzed with Tukey’s test.

To elucidate the effects of 20S on the osteogenic differentiation of MSCs, we examined the effects of oxysterol on osteogenic differentiation of MSCs through cell cytochemistry and gene expression analysis. Cells treated with 20S induced a higher proportion of mineralization markers, Alizarin Red (Figure 1) and Von Kossa stain (Figure 2), compared to cells treated with OM and C. Moreover, 10 µM of 20S significantly increased bone sialoprotein (BSP), bone morphogenic protein (BMP2), collagen type I α 2 chain (Col1A2), sonic hedgehog (Shh), and runt-related transcription factor 2 (RunX2) expression compared to other treatment groups at 24 h (Figure 3A,B,D,G and H. The expression of Gli 1 was higher in cells treated with 10 µM 20S compared to control cells and cells treated with osteogenic media at all time points of the study (Figure 3C). At 24 h, a significant increase in patched (Ptch) mRNA expression was observed in cells treated with OM and all levels of 20S compared to control cells (Figure 3E). However, the inclusion of 20S did not increase Pthc expression compared to cells treated with OM. At 72 h and 7 d, cells treated with 10 µM of 20S significantly increased Pthc mRNA expression compared to cells in C or OM treatments (Figure 3E). At 7 d, bone γ-carboxyglutamic acid-containing protein (BGLAP) mRNA expression was significantly increased in cells treated with 10 µM of 20S (Figure 3F). OM and all levels of 20S significantly increased mRNA expression of Shh. However, there was no significant difference between cells treated with OM only or 20S (Figure 3G). Results indicated that 10 µM 20S could induce a stimulatory effect on the osteogenic differentiation of cBMSCs.

### 3.2. Role of Hh Signaling in Osteogenic Differentiation of cBMSCs

**Figure 4:**
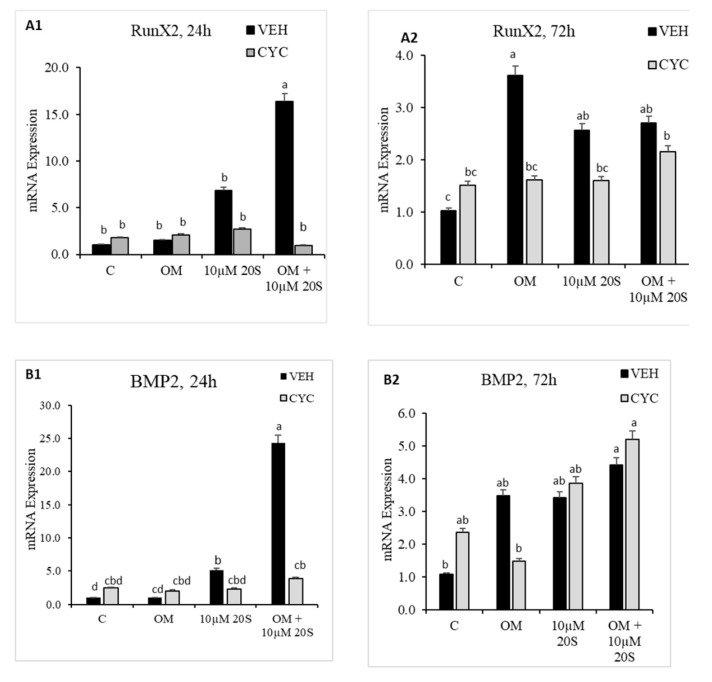

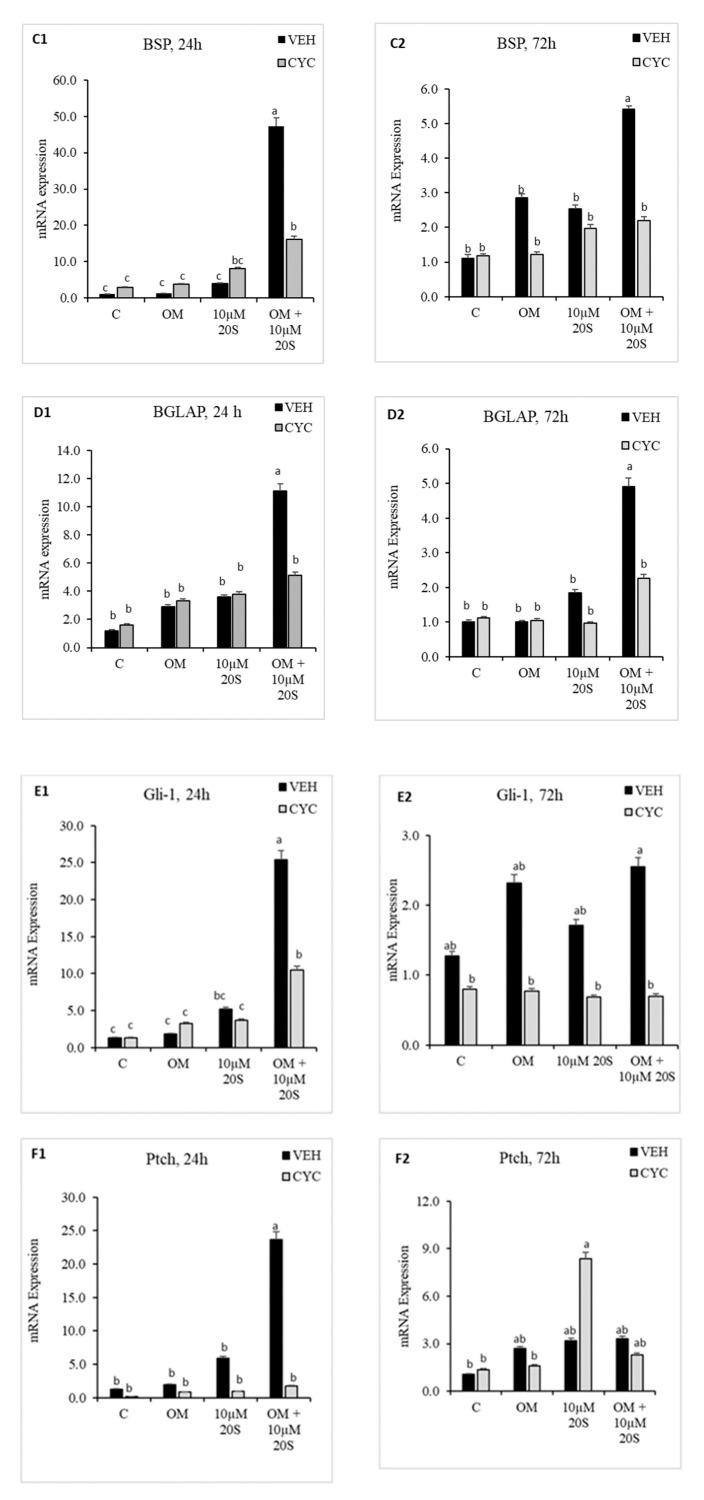

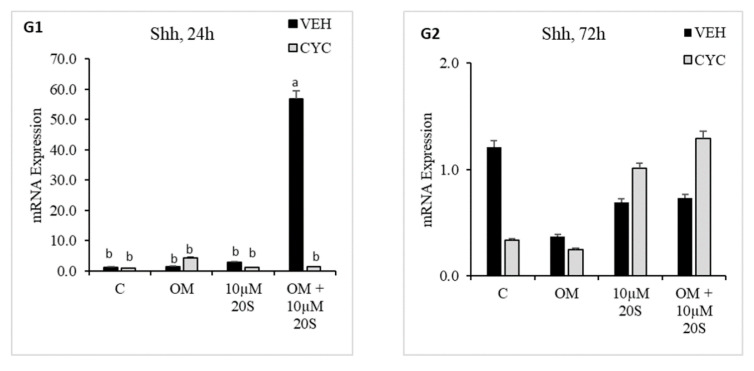
20S activates osteogenic genes through the Hedgehog signaling pathway. Confluent chicken bone marrow stem cells (cBMSCs) were pretreated for 2 h with control vehicle (VEH) or 4 µM cyclopamine (CYC), followed by 24 h and 72 h of treatment with (1) control vehicle (**C**), (2) osteogenic media (OM) containing DMEM with 10% FBS, 50 µg/mL ascorbate, 0.5 µM DXA, and 10 mM β-glycerophosphate, (3) 10 µM 20S, or (4) OM + 10 µM 20S. RNA was isolated, reverse transcribed to cDNA, and qRT-PCR was conducted to analyze osteogenic gene expression. GAPDH was used as a housekeeping gene. Fold changes in gene expression relative to the control were calculated using the −ΔΔCt method. mRNA expression of (**A1**,**A2**) RunX2, (**B1**,**B2**) BMP2, (**C1**,**C2**) BSP, (**D1**,**D2**) BGLAP, (**E1**,**E2**) Gli-1, (**F1**,**F2**) Ptch, and (**G1**,**G2**) Shh were significantly reduced in cells treated with OM + 20S in the Cyc pretreated group compared to the VEH pretreated group. Bars represent mean ± SEM of triplicate determinations. Bars without a letter (a–b) in common are significantly different (*p* < 0.05) when analyzed with Tukey’s test.

To determine whether an Hh signaling mechanism is involved in regulating osteogenic differentiation in MSCs by oxysterol, 20S was added to the cBMS cells pretreated with vehicle only or cyclopamine solution. The expression of osteoblastic differentiation markers were examined 24 h and 72 h after treatment with 20S. Vec pretreated cells treated with 20S + OM had significantly increased mRNA expression of Hh target genes, Gli, Shh, and Pthc, at 24 h (Figure 4E–G). mRNA expression of other osteogenic genes, RunX2, BMP2, BSP, and BGLAP, were also significantly increased in vehicle pretreated cells treated with OM + 20S compared to control mediaat both 24 h and 72 h of treatment (Figure 4A–D). Cyclopamine completely reversed the effect of OM + 20S on the expression of osteogenic differentiation marker genes, RunX2, BMP2, BSP, BGLAP, Gli, Ptch, and Shh, at 24 h post-treatment (Figure 4(A1)–(G1)). Cyclopamine continued to block mRNA expression of BSP, BGLAP, and Gli1 in cells treated with OM + 20S up to 72 h of treatment (Figure 4(C2)–(E2)).

### 3.3. Effects of 20S on Adipogenic Differentiation of cBMSCs

**Figure 5:**
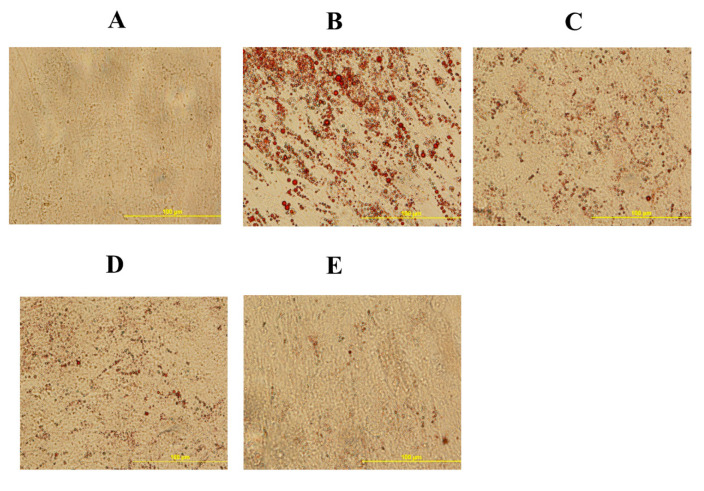
20S inhibits adipogenic differentiation of mesenchymal stem cells isolated from compact bones of chicken. Cells were treated with (**A**) Control, (**B**) Oleic acid media (OA), (**C**) OA + 2.5 µM 20S, (**D**) OA + 5 µM 20S, or (**E**) OA + 10 µM 20S for 96 h. Adipocyte formation was examined by Oil Red O staining procedure. Pictures were taken by Olympus DP70 at 20X.

**Figure 6:**
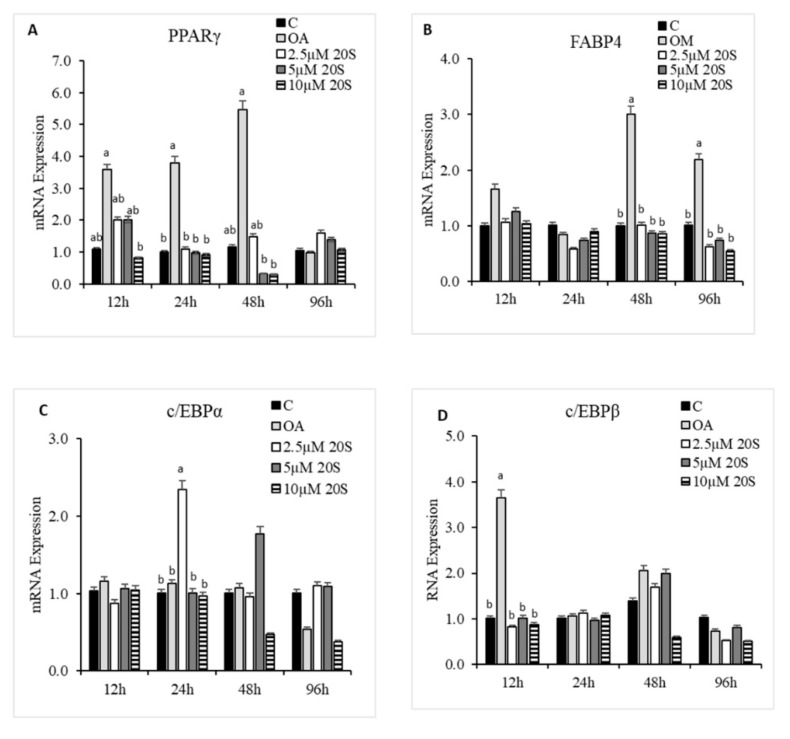

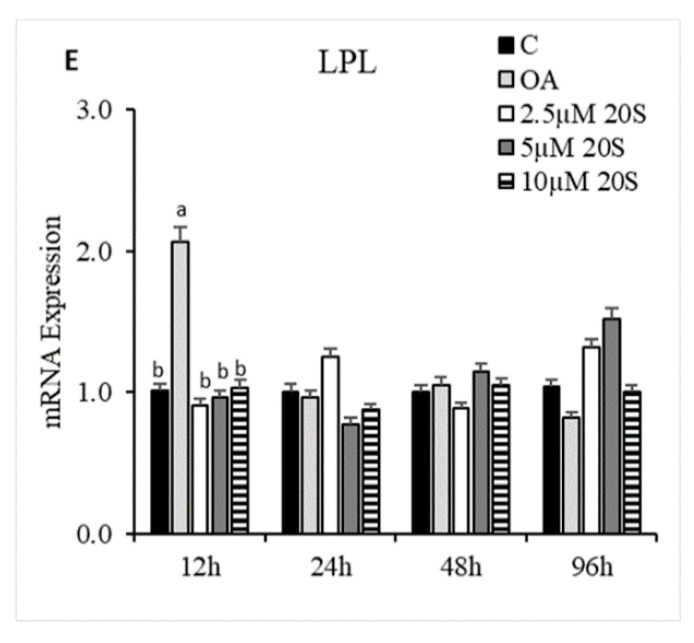
Adipogenic effects of 20S oxysterol on mesenchymal stem cells. Relative mRNA expression levels of osteogenic genes (**A**) PPARγ, (**B**) FABP4, (**C**) c/EBPα, (**D**) c/EBPβ, and (**E**) LPL in compact bone-derived mesenchymal stem cells in chicken (cBMSCs) treated with different levels of oxysterol. cBMSCs were treated with (1) Control (C), (2) Oleic acid media (OA), (3) OA + 2.5 µM 20S, (4) OA + 5 µM 20S, or (5) OA + 10 µM 20S. Cells were harvested at 12 h, 24 h, 48 h, and 96 h after treatment. RNA was isolated, reverse transcribed to cDNA, and qRT-PCR was conducted to analyze osteogenic gene expression. GAPDH was used as a housekeeping gene. Fold changes in gene expression relative to the control were calculated using the −ΔΔCt method. Bars represent mean ± SEM of triplicate determinations. For each harvest period, bars without a letter (a–b) in common within the harvested period are significantly different (*p* < 0.05) when analyzed with Tukey’s test.

To understand the effect of oxysterol in adipogenic differentiation of cBMSCs, we examined the effects of adipogenic genes on mRNA expression in cells treated with different doses of 20S in adipogenic media. Cells treated with OA had increased adipocyte formation as detected by oil red O staining and quantified using a light microscope. In contrast, the inclusion of 20S decreased adipocyte formation (Figure 5). Compared to control cells, cells treated with OA alone had significantly increased mRNA expression of peroxisome proliferator-activated receptor γ (PPARγ), fatty acid-binding protein (FABP4), lipoprotein lipase (LPL), and CCAAT/enhancer binding protein β (C/EBPβ) at different time points during the study. OA induced expression of PPARγ expression at 12 h, 24 h, and 48 h of treatment (Figure 6A), FABP4 expression at 48 h and 96 h of treatment (Figure 6B), and C/EBPβ (Figure 6D) at 12 h of treatment. Inclusion of oxysterol decreased OA-induced adipogenic mRNA expression in cBMSCs at different time points during the study. PPARγ mRNA expression was significantly reduced in cells treated with all three levels of 20S compared to OA-treated cells at 12, 24, and 48 h of treatment (Figure 6A). Cells treated with 10 µM of oxysterol had significantly decreased mRNA expression of FABP4 compared to cells treated with OA for 48 and 96 h of treatment (Figure 6B). Cells treated with 10 µM 20S had considerably reduced mRNA expression of C/EBPβ at 12 h and 48 h compared to cells treated with OA alone (Figure 6D). Results indicated that the inclusion of 20S could reduce the adipogenic differentiation of cBMSCs.

### 3.4. Role of Hh Signaling Mechanism in Adipogenic Signaling Pathway

**Figure 7:**
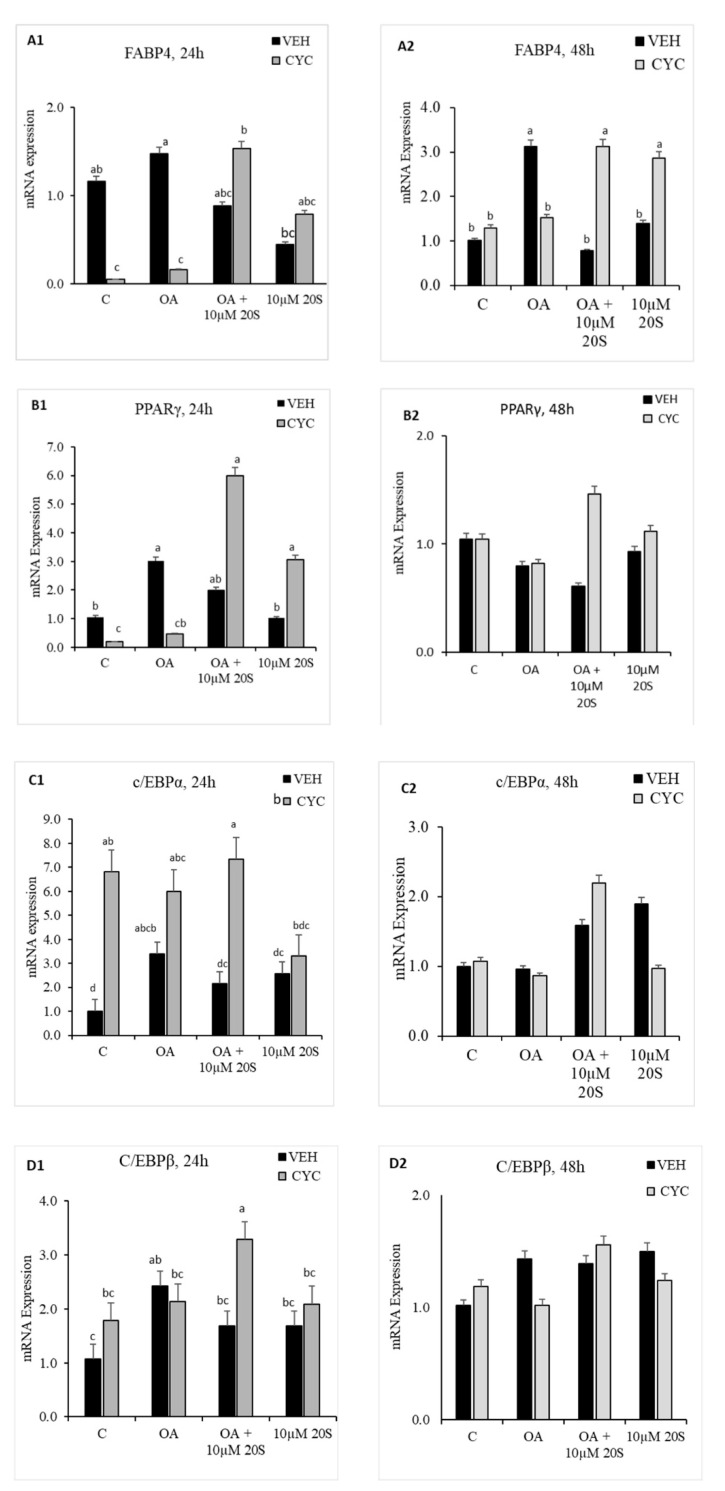

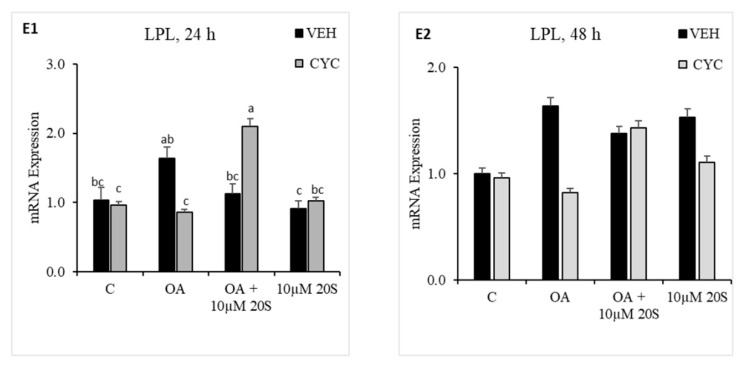
Hedgehog signaling pathway inhibitor, Cyclopamine, blocked the inhibitory effect of 20S on OA-induced adipogenic differentiation of chicken-bone marrow stem cells (cBMSCs). When confluent, cBMSCs were pretreated for 2 h with control vehicle (VEH) or 4 µM cyclopamine (CYC) followed by 24 h and 48 h of treatment with (1) control vehicle (**C**), (2) Oleic acid (OA), (3) OA + 10 µM 20S, or (4) 10 µM 20S. RNA was isolated, reverse transcribed to cDNA, and q RT-PCR was conducted to analyze osteogenic gene expression. GAPDH was used as a housekeeping gene. Fold changes in gene expression relative to the control were calculated using the −ΔΔCt method. mRNA expression of (**A1**,**A2**) FABP4, (**B1**,**B2**) PPARγ, (**C1**,**C2**) c/EBPα, (**D1**,**D2**) c/EBPβ, and (**E1**,**E2**) LPL are presented above. Data from the representative experiment are reported as the mean of the triplicate determination. Bars represent mean ± SEM for triplicate determinations. Bars without a letter (a–b) in common are significantly different (*p* < 0.05) when analyzed with Tukey’s test.

In response to previous reports regarding mouse and human MSCs about the importance of the hedgehog signaling mechanism of oxysterol in osteogenic differentiation and anti-adipogenic potential [40], we examined whether Hh signaling pathway played a role in the anti-adipogenic effects of 20S in cBMSCs. OA-treated cells had significantly increased expression of FABP4 (Figure 7(A2)), PPARγ (Figure 7(B1)), and CEBPβ (Figure 7(D1)) at a different time points during the study. Inclusion of 20S blocked the mRNA expression of FABP4 (Figure 7(A2)), PPARγ (Figure 7(B1)), and CEBPβ (Figure 7(D1)) in the cells harvested at the same time points. Cells pretreated with cyclopamine had significantly increased expression of FABP4 (Figure 7(A2)), PPARγ (Figure 7(B1)), CEBPβ (Figure 7(D1)), and LPL (Figure 7(E1)) at 24 h and FABP4 (Figure 7(A2)) at 48 h, which was blocked by 20S treatment in the Veh pretreated group (Figure 7). This study indicates that treatment with cyclopamine completely abolished the inhibitory effects of 20S and increased adipogenic mRNA gene expression of FABP4, c/EBPβ, PPARγ, and LPL at 24 h and mRNA expression of FABP4 and PPARγ at 48 h of treatment.

### 3.5. Effect of 20S in Myogenic Differentiation of cBMSCs

**Figure 8:**
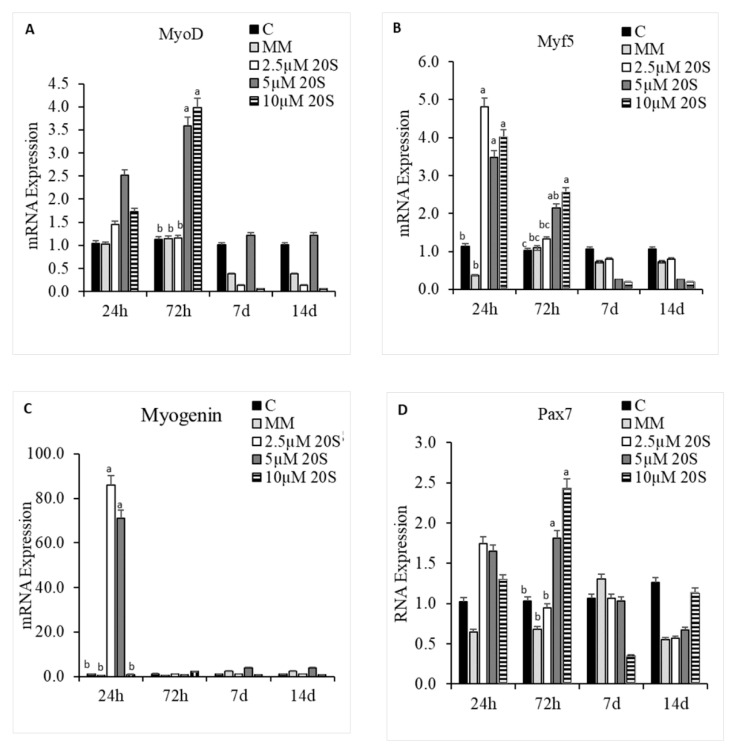
Myogenic effects of 20S oxysterol in mesenchymal stem cells. Relative levels of mRNA expression of osteogenic genes, (**A**) MyoD, (**B**) Myf5, (**C**) Myogenin, and (**D**) Pax7, in compact bone-derived mesenchymal stem cells in chicken (cBMSCs) treated with different levels of oxysterol. cBMSCs were treated with (1) Control (C), (2) myogenic media (MM) containing DMEM with 5% Horse Serum, 10 µM Hydrocortisone, and 0.1 µM DXA, (3) MM + 2.5 µM 20S, (4) MM + 5 µM 20S, or (5) MM + 10 µM 20S. Cells were harvested at 24 h, 72 h, and 7 d after treatment. RNA was isolated, reverse transcribed to cDNA, and qRT-PCR was conducted to analyze osteogenic gene expression. GAPDH was used as housekeeping genes. Fold changes in gene expression relative to the control were calculated using the −ΔΔCt method. Bars represent mean ± SEM of triplicate determinations. Within each harvest period, bars without a letter (a–b) in common are significantly different (*p* < 0.05) when analyzed with Tukey’s test.

In addition to the effects of 20S on osteogenic and adipogenic differentiation of cBMSCs, we examined the effect of 20S on the myogenic differentiation potential of cBMSCs. 5 µM and 10 µM of 20S increased MyoD mRNA expression at 72 h of treatment compared to control cells (Figure 8A). Inclusion of 20S oxysterol increased mRNA expression of myogenic factor 5 (Myf5) compared to the control cells at 24 h and 72 h of treatment (Figure 8B). 2.5 µM and 5 µM of 20S increased mRNA expression of Myogenin compared to control cells at 24 h of treatment. Cells treated with 5 µM and 10 µM of 20S had significantly increased mRNA expression of paired box 7 (Pax7) compared to the control cell at 72 h of treatment. However, the expression of myogenic genes treated with oxysterol was decreased to the basal level by 7 d of the study.

### 3.6. Role of Hh Signaling Mechanism in Myogenic Signaling Pathway

**Figure 9:**
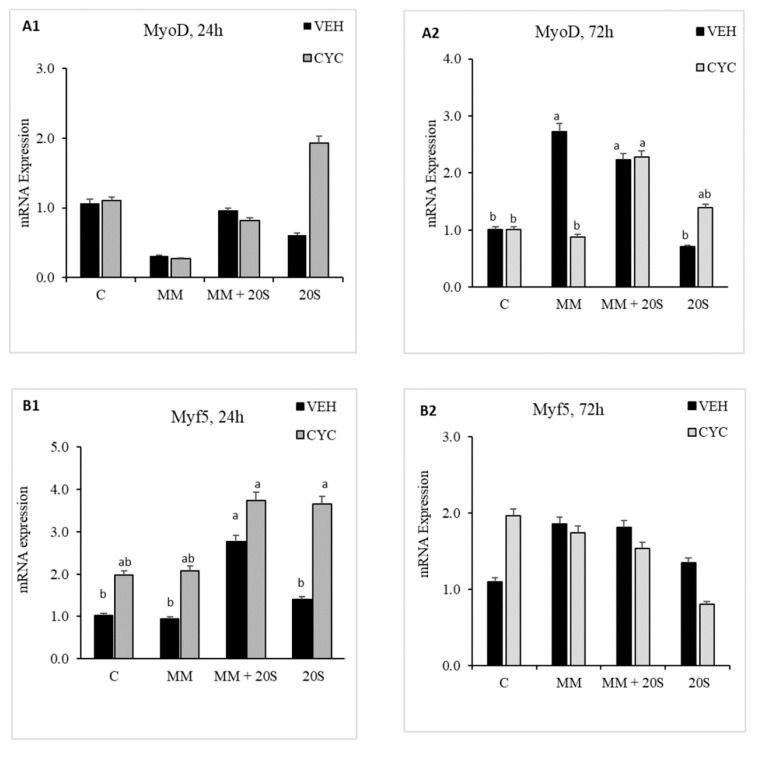

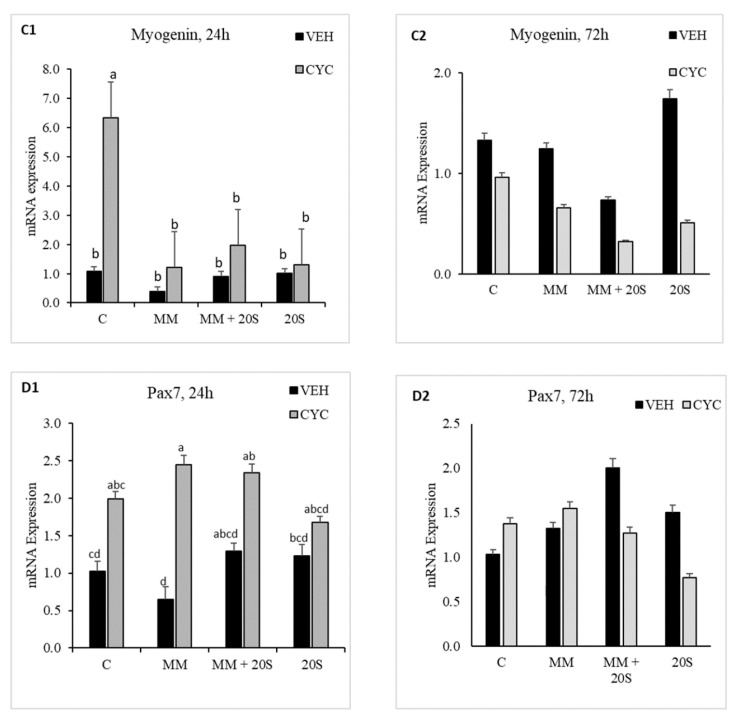
Hedgehog signaling pathway inhibitor, Cyclopamine, did not block the inhibitory effect of 20S on myogenic differentiation of chicken bone marrow stem cells (cBMSCs). When confluent, cBMSCs were pretreated for 2 h with control vehicle (VEH) or 4 µM cyclopamine (CYC), followed by 24 h and 72 h of treatment with (1) Control (C), (2) myogenic media (MM) containing DMEM with 5% Horse Serum, 10 µM Hydrocortisone, and 0.1 µM DXA, (3) MM + 2.5 µM 20S, (4) MM + 5 µM 20S, and (5) MM + 10 µM 20S. RNA was isolated, reverse transcribed to cDNA, and q RT-PCR was conducted to analyze osteogenic gene expression. GAPDH was used as a housekeeping gene. Fold changes in gene expression relative to the control were calculated using the −ΔΔCt method. mRNA expression of (**A1**,**A2**) MyoD, (**B1**,**B2**) Myf5, (**C1**,**C2**) Myogenin, and (**D1**,**D2**) Pax7 are presented above. Bars represent mean ± SEM of triplicate determinations. Bars without a different letter (a–b) in common are significantly different (*p* < 0.05) when analyzed with Tukey’s test.

To determine whether Hh signaling pathway was involved in oxysterol-induced myogenic differentiation of cBMSCs, cells were pretreated with Cyc or Veh for 2 h before being subjected to myogenic media and 20S. In cells subjected to a combination of myogenic media and oxysterol, there was no significant difference in mRNA expression of MyoD (Figure 9(A1,A2)), Myogenin (Figure 9(B1,B2)), Myf5 (Figure 9(C1,C2)), or Pax7 (Figure 9(D1,D2)) between cells pretreated with or without cyclopamine. However, compared to pretreatment with Veh, pretreatment with cyclopamine increased expression of Pax7 mRNA at 24 h in cells treated with MM alone and expression of Myf5 mRNA in cells treated with 20S alone. Pretreatment with cyclopamine decreased mRNA expression of MyoD at 72 h compared to Veh-pretreated cells treated with myogenic media only. This study indicates that oxysterol-induced myogenic expression of cBMSCs is not mediated with the Hh signaling pathway.

## 4. Discussion

Primary MSCs have multilineage capacity and can differentiate to osteogenic, adipogenic, and myogenic lineages dependent on the specific cues they are subjected to. In this study, osteogenic media, adipogenic media, and myogenic media induced cBMSCs to osteogenic, adipogenic, and myogenic differentiation of cells, respectively, indicating that primary cells cultured from the compact bone in chicken are capable of multilineage differentiation. MSCs isolated from mouse and human compact bones have similar multilineage differentiation capacities. They also easily adhere to plastic surfaces [19,51]. Multilineage differentiation potential and ability to adhere to plastic surfaces under standard culture medium are two of the three minimum criteria that The Mesenchymal and Tissue Stem Cell Committee of the International Society for Cellular Therapy (ISCT) has published for the cells to be defined as MSCs [52].

The present study demonstrates that 20S can induce osteogenic and myogenic differentiation and reduce the adipogenic differentiation of cBMSCs. This is the first study conducted to understand oxysterol’s ability to regulate osteogenic, adipogenic, and myogenic differentiation of chicken-derived stem cells. Our data strongly suggest that 10 µM 20S can induce osteogenic differentiation of cBMSCs. Detection of mineralization of osteoblasts via the Alizarin red and Von Kossa assay confirms cellular commitment towards the osteoblastic differentiation pathway and provides clear evidence of functional, mature cells [48,53]. In our study, the same assay demonstrated that cBMSCs treated with 10 µM of 20S had higher deposition of calcium than control cells and cells treated with OM alone. Such mineral deposits begin during extracellular matrix maturation and indicate the late stages of osteogenic differentiation of MSCs [41].

In the present study, 10 µM of 20S increased mRNA expression of osteogenic genes such as BSP, BMP2, col1A2, and RunX2. Run X2 is a key osteogenic gene that indicates the early commitment of MSCs to osteochondrogenic progenitor cells. RunX2 directs MSCs to differentiate into pre-osteoblast and inhibits adipogenic and chondrogenic differentiation, thereby maintaining a supply of immature osteoblasts. [54]. Immature osteoblasts express a high level of osteopontin whereas BGLAP, Col1a1, and BSP are measured in high levels when differentiated to mature osteoblasts. [55]. The mature (induced) osteoblasts show positive Von Kossa stain and Alizarin Red assay in vitro [55] as found above.

Our findings regarding mineralization and osteogenic gene expression indicate that oxysterol is a potent bioactive molecule to induce osteogenic differentiation in cBMSCs. These results agree with detailed studies of oxysterol in mouse, rabbit, and rat. A combination of 2 naturally occurring oxysterols, 20S and 22S, induced osteoinductive effects in pluripotent mouse marrow stromal cell line M2-10B4 [56,57]. Similarly, oxysterol 133 induced expression of RunX2, ALP, BSP, and BGLAP mRNA in C3H10T1/2 mouse embryonic fibroblasts and M2-10B4 mouse marrow stromal cells [58]. In the same study, oxysterol 133 increased bilateral posterolateral lumbar spinal fusion of L4-L5 in Lewis rats whose transverse process was decorticated with high-speed burr. Similarly, oxysterol 49, a novel oxysterol analog, induced osteogenic properties similar to BMP2 in rabbit bone marrow stromal cells [59].

Apart from oxysterol’s osteogenic property, it is reported that oxysterol also possesses anti-adipogenic effects in mouse and human pluripotent mesenchymal stem cells [40]. In this study, OA significantly increased PPARγ and c/EBPβ as early as 12 h post treatment, which then increased the expression of FABP4 at 48 h and 96 h. OA has been reported to increase adipogenic differentiation in chicken-derived MSCs rather than using DMI alone, an adipogenic cocktail used in mammalian cells differentiation [60,61]. In our study, the inclusion of 20S with OA in the cBMSCs suppressed the expression of PPARγ, c/EBPβ, and aP2 mRNA, expressions induced by OA alone. This indicates that PPARγ and c/EBPβ could regulate the adipogenic differentiation of cBMSCs, and the use of oxysterol could have suppressed the adipogenic differentiation by suppressing the effect of PPARγ or c/EBPβ. It has been previously reported that Novel oxysterol compounds have the potential to inhibit adipocyte differentiation and decrease expression of adipogenic marker genes such as PPARγ in mouse and human-derived progenitor cells [39,40]. PPARγ is previously described as a key regulator of adipogenic differentiation [62]. In early adipogenesis, expression of c/EBPβ and c/EBPδ induced the expression of PPARγ and c/EBPα which then regulate the positive feedback mechanism that induces other genes for adipogenic differentiation. PPARγ is thought to also play a role in the regulation of bone metabolism. Embryonic stem cells derived from homozygous PPARγ -deficient mice failed to show adipogenic differentiation when treated with troglitazone, whereas the expression of RunX2 and osteocalcin gene were remarkably increased [63]. Heterozygous PPARγ -deficient mice did exhibit increased osteogenesis of cortical and trabecular bone. They had decreased adipocyte differentiation compared to wild types in vivo and in the bone marrow progenitors of those mice in vitro [63,64]. However, when a PPARγ expression construct was transfected to murine-derived bone marrow stromal cells and activated with hiazolidinones, the cell lines increased adipocyte differentiation. The transfer decreased the expression of RunX2 and osteocalcin [65]. In combination, these results indicate that PPARγ could be a key player in the adipogenesis of cBMSCs. The use of oxysterol could have suppressed adipogenic differentiation of cBMSCs by suppressing the expression of PPARγ.

Because our results demonstrated that 20S is a novel oxysterol compound that can increase osteogenic and decrease adipogenic differentiation of cBMSCs, we investigated the molecular mechanisms included and whether the Hh signaling pathway is involved. It has been reported that oxysterol is a novel activator of Hh signaling pathway to influence the osteogenic and adipogenic differentiation of mouse- and human-derived MSCs [56]. Our data provided several lines of evidence that 20S could direct the osteoinductive and anti-adipogenic effects through activation of hedgehog signaling pathway. cBMSCs treated with 20S along with OM demonstrated an increase in mRNA expression of Hh target markers, such as Gli1, Ptch, and Shh. In addition, mRNA expression of osteogenic markers, such as Gli1, Pthc, BSP, RunX2, BGLAP, and BMP2, were reduced in in cBMSCs induced by 20S, but subjected to hedgehog pathway inhibitor, cyclopamine pretreatmentcompared to the mRNA expression of cBMSCs pre-treated with Veh alone. Cyclopamine continued to suppress osteogenic differentiation markers’ expression until 72 h of the study, which suggests that the activation of Hh signaling pathway is prominent in the pro-osteogenic mechanism by which 20S regulates osteogenic differentiation of MSCs.

To elucidate the molecular mechanism by which 20S inhibits the expression of adipogenic genes expressed in cBMSCs when treated with OA, we focused on Hh signaling pathway. The use of Hh signaling pathway inhibitor, cyclopamine, on cells treated with OA and 20S reversed the normal inhibitory effects of 20S and induced expression of adipogenic genes PPARγ, c/EBPβ, and aP2. These results indicate that 20S could block PPARγ promoter activity stimulated by C/EBPβ through Hh signaling mechanism. Consistent with our findings in cBMSCs, 20S has been reported to inhibit expression of PPARγ and adipogenic differentiation of M2-10B4 murine pluripotent bone marrow stromal cell lines through Hh signaling-dependent mechanism [39]. Similarly, Sonic Hedgehog (Shh) signaling pathway was reported to reduce the adipogenic differentiation of C3H10T1/2 pre-adipocyte cells by decreasing the expression of c/EBPα and PPARγ transcription factors [66].

Hedgehog signaling pathway plays a key role in various embryonic development, growth, and patterning of tissues, postnatal development and maintenance of tissue/organ integrity and function, stem cell physiology, cancer, and cardiovascular disease [41]. There are three members of Hh family: Indian Hh, Sonic Hh, and Desert Hh. Hh signaling involves a complex array of factors that influences the function and distribution of Hh molecules. Hedgehog signaling begins with the binding of hedgehog protein to a transmembrane protein Patch, which removes the inhibitory effects on another transmembrane protein, Smo, in Hh responsive cells [67]. Smo activates the intracellular signaling cascade, resulting in activation of Gli transcription factors, which transcribe the Hh target genes, Gli-1 and Ptch [56]. This transcription regulation involves the Ci/Gli transcription factors and intricate interaction among membranes of a complex accessory molecule including Fused, suppressor of fused, and Rab 23 to regulate localization and stability of Gli [67]. Previous experiments in mice reported that lacking Indian Hh resulted in the disorder of endochondral bone patterning and osteoblast formation. Those mice lacking sonic hedgehog showed disorders in craniofacial bones, vertebral column, and calcified ribs [68,69]. Previous studies have also reported that the lineage-specific osteogenic differentiation of MSCs is controlled by Hh signaling mechanism [70,71]. Furthermore, there are several reports of other bioactive compounds that regulate osteogenic and adipogenic differentiation of MSCs derived from humans and mice through Hh signaling [72,73].

Previous studies have reported that different forms of oxysterol, such as oxy 49, oxy 133, 20S, and 22S induced osteogenic expression and reduced adipogenic expression potential of MSCs derived from mouse and human cells through Hh signaling mechanism [39,41,53,56,58,74,75]. Further studies in mouse and human MSCs have indicated that oxysterol could induce osteogenic differentiation of mouse and human stromal cells through other pathways: a Wnt signaling related, Dkk-1-inhinitable and PI3-kinase mechanism [76], activation of HES-1 and HEY-1 expression, and LXR activation pathway [41]. Studies in mouse NIH-3T3 fibroblast, C3H10T1/2 MSCs, and 3T3-L1 preadipocytes have shown that Hh signaling blocks the early step of adipogenesis upstream of PPARγ, presumably after mitotic clonal expansion, by regulating Gata expression [77]. This indicates that the anti-adipogenic effect through Hh signaling could be regulated at least in part by Gata expression.

Several in vitro studies have demonstrated that, in vitro, MSCs can interconvert between osteoblast and adipocyte phenotypes [78]. In addition, it has been reported that, under appropriate conditions, mature osteoblasts can undergo adipogenesis [30] and mature adipocytes have been differentiated along the osteoblastic pathway. Several studies demonstrate an inverse relationship between osteoblast and adipocyte differentiation. TAZ increased the transcription of RunX2-dependent gene and decreased expression of PPARγ, thus promoting osteogenesis and reducing adipogenesis [79]. Similarly, Dlk1 has been reported to be involved in the regulatory effects of both osteoblast and adipocyte differentiation of human-derived MSCs [80]. It has also been described that oxysterols affect cyclo-oxygenase and phospholipase A2 and act in synergy with bMP2 in inhibiting adipogenesis and inducing osteogenic differentiation [40]. It has been described that 20S could cause a pro-osteogenic effect on MSCs by Hh signaling through activating expression of Notch target genes Hes-1, HEY-1, and HEY-2 in bone marrow stem cells [41].

Notch target genes are involved in various biological processes, including osteogenesis, adipogenesis, and myogenesis. Our study demonstrated that 20S oxysterol significantly increased myogenic expression of MyoD, Myogenin, MyF5, and Pax7 mRNA expression. A previous study in human umbilical cord-derived MSCs showed MyoD and Myogenin were expressed as early as 3 days after incubation with myogenic media, but were not expressed after two weeks of culture [81]. Myogenic differentiation of MSCs occurs via activation of myogenic transcription factors: PAX3, MyoD, Myf-5, and Myogenin [82,83]. Pax3 and Pax7 are master regulators of myogenic differentiation and contribute to early striated muscle development during skeletal muscle differentiation [84]. cBMSCs pretreated with the Hh signaling inhibitor cyclopamine, did not show a decrease in the expression of myogenic transcription factors indicating that Hh signaling did not play a role in controlling myogenic regulation by 20S oxysterol.

Myogenesis is regulated by a family of transcription factors, including MyoD, Myogenin, Myf5, and MRF4 [85,86]. Skeletal myogenesis is the developmental cascade that involves the regulatory MyoD gene family that determines the progress of multipotent mesodermal stem/progenitor cells into myogenic lineage [87]. Skeletal muscle stem cells are composed of multinucleated myofibers established during embryogenesis by the fusion of myogenic cells, also called satellite cells [88]. Satellite cells are considered as precursors for muscle growth and repair. Typically, the myofiber nuclei are mitotically inactive, but they are activated to respond to muscle damage and provide progeny cells for myofiber repair and growth. Thus, satellite cells exhibit stem cell-like properties and are competent to form the basal origin of muscle regeneration [89]. Myogenesis of satellite cells is regulated by muscle-specific regulatory factors such as MyoD, Myf5, and Myogenin. Mouse satellite cells and myogenic cell lines express Myf5 protein in proliferating cells, whereas the protein was not detected upon differentiation and fusion with myotubes [90]. The expression of MyoD is observed during satellite-cell proliferation, whereas their differentiation is marked by the expression of Myogenin with a decrease in PAx7 and Myf5 [90,91]. Skeletal myogenesis is a developmental cascade, where MyoD family and myogenic regulator factors directly regulate myocyte cell specification, differentiation, and expression at the early stage of myogenic differentiation [81,86]. Mutant mice that lack MyoD and dystrophin displayed a significant increase in the severity of myopathy and premature death, highlighting the role of MyoD in muscle regeneration [84]. In a previous study, the treatment of muscle cells derived stem cells indicates that incubation of satellite cells with 1-25-D3 increased myogenic markers such as MyoD, Myogenin, MYH1, and muscle troponin [65].

## 5. Conclusions

In summary, this study shows that 20S is an oxysterol compound with pro-osteogenic, pro-myogenic, and anti-adipogenic properties in cBMSCs. Our study further provides evidence that 20S increased the osteogenic differentiation and decreased adipogenic differentiation of cBMSCs. And the actions of 20S were involved inHh signaling pathway. Furthermore, 20S oxysterol also increased the myogenic differentiation of cBMSCs, but did not exert its differentiation through Hh signaling pathway. These findings provide evidence that oxysterols could induce the Hh signaling pathway and, therefore, could play an important role in other developmental processes in chicken. Further study needs to be conducted to improve our understanding of the detailed molecular mechanisms and downstream targets of oxysterol in cBMSCs. This research could lead to novel oxysterol compounds and intervention therapies targeting MSCs regeneration/lineage differentiation to tackle skeletal-, adipose-, and muscle-derived problems in poultry with economic and welfare implications for the industry.

## Figures and Tables

**Table 1:** Primers sequences that were used for qRT-PCR gene expression assay.

Gene	Primer Sequence (5′-3′)	Product Length (bp)	Annealing Temperature (°C)	Accession #
GAPDH	Fwd: GCTAAGGCTGTGGGGAAAGT	161	55	[49]
	Rev: TCAGCAGCAGCCTTCACTAC			
BSP	Fwd: CAGTGGGAGTACGAGGTGAC	141	55	NM_205162.1
	Rev: CAGTGGGAGTACGAGGTGAC			
Gli1	Fwd: GCACAGCTCCAACGACCGCT	205	57	NM_001305245.1
	Rev: GTTGCCGTCGGAAGCACCCA			
BMP2	Fwd: TCAGCTCAGGCCGTTGTTAG	163	57	NM_204358.1
	Rev: GTCATTCCACCCCACGTCAT			
BGLAP	Fwd: GACGGCTCGGATGCTCGCAG	226	56	[49]
	Rev: CAGACGGGGCCGTAGAAGCG			
Ptch	Fwd: GGCGTTCGCGGTGGGACTAC	205	56	NM_204960.2
	Rev: GGTGCTGCCGGAGTGCTTCT			
Shh	Fwd: TGC TAG GGA TCG GTG GAT AG	197	56	NM_204821.1
	Rev: ACA AGT CAG CCC AGA GGA GA			
RunX2	Fwd: ACTTTGACAATAACTGTCCT	192	52	NM_204821.1
	Rev: GACCCCTACTCTCATACTGG			
FABP4	Fwd: TGCTGGGCATCTCAATCACA	106	57	[49]
	Rev: GCATTAGTCAGAACGGGCCT			
PPARγ	Fwd: TGAATGTCGTGTGTGTGGGG	229	55	[49]
	Rev: GCATTCGCCCAAACCTGATG			
C/EBPα	Fwd: CCTACGGCTACAGAGAGGCT	205	55	[49]
	Rev: GAAATCGAAATCCCCGGCCA			
C/EBPβ	Fwd: CCGCTCCATGACCGAACTTA	204	55	[49]
	Rev: GCCGCTGCCTTTATAGTCCT			
LPL	Fwd: TGCCCCTATCCGCCTCTCCC	297	57	[49]
	Rev: GTTGCAGCGGTAGGCCATGCT			
Col1A2	Fwd: AGAAAGGAATCCAGCCCAAT	237	58	NM_204426.1
	Rev: ACACCTGCCAGATTGATTCC			
MyoD	Fwd: CAGCAGCTACTACACGGAATCA	102	57	[50]
	Rev: GGAAATCCTCTCCACAATGCTT			
Myogenin	Fwd: AGCAGCCTCAACCAGCAGGA	179	58	NM_204184.1
	Rev: TCTGCCTGGTCATCGCTCAG			
Pax7	Fwd: AGGCTGACTTCTCCATCTCTCCT	156	57	XM_015296832.1
	Rev: TGTAACTGGTGGTGCTGTAGGTG			
Myf5	Fwd: GAGGAACGCCATCAGGTACATC	126	57	NM_001030363.1
	Rev: ACATCGGAGCAGCTGGAGCT			
